# The SycN/YscB chaperone-binding domain of YopN is required for the calcium-dependent regulation of Yop secretion by *Yersinia pestis*

**DOI:** 10.3389/fcimb.2013.00001

**Published:** 2013-01-24

**Authors:** Sabrina S. Joseph, Gregory V. Plano

**Affiliations:** ^1^Department of Microbiology and Immunology, F. Edward Hérbert School of Medicine, Uniformed Services University of the Health SciencesBethesda, MD, USA; ^2^Department of Microbiology and Immunology, University of Miami Miller School of MedicineMiami, FL, USA

**Keywords:** type III secretion, chaperone, bacterial pathogenesis, *Yersinia pestis*, plague

## Abstract

Numerous Gram-negative bacterial pathogens employ type III secretion systems (T3SSs) to inject effector proteins into eukaryotic cells. The activation of the type III secretion (T3S) process is tightly controlled in all T3SSs. In *Yersinia pestis*, the secretion of effector proteins, termed *Yersinia* outer proteins (Yops), is regulated by the activity of the YopN/SycN/YscB/TyeA complex. YopN is a secreted protein that interacts with the SycN/YscB chaperone via an N-terminal chaperone-binding domain (CBD) and with TyeA via a C-terminal TyeA-binding domain (TBD). Efficient YopN secretion is dependent upon its N-terminal secretion signal (SS), CBD, and the SycN/YscB chaperone. In this study, we investigate the role of the YopN CBD in the regulation of Yop secretion. Analysis of YopE/YopN hybrid proteins in which the YopN SS or SS and CBD were replaced with the analogous regions of YopE indicated that the YopN CBD or SycN/YscB chaperone play a role in the regulation of Yop secretion that is independent of their established roles in YopN secretion. To further analyze the role of the YopN CBD in the regulation of Yop secretion a series of tetra-alanine substitution mutants were generated throughout the YopN CBD. A number of these mutants exhibited a defect in the regulation of Yop secretion but showed no defect in YopN secretion or in the interaction of YopN with the SycN/YscB chaperone. Finally, conditions were established that enabled YopN and TyeA to regulate Yop secretion in the absence of the SycN/YscB chaperone. Importantly, a number of the YopN CBD mutants maintained their defect in the regulation of Yop secretion even under the established SycN/YscB chaperone-independent conditions. These studies establish a role for the CBD region of YopN in the regulation of Yop secretion that is independent from its role in YopN secretion or in the binding of the SycN/YscB chaperone.

## Introduction

Numerous Gram-negative bacterial pathogens use type III secretion systems (T3SSs) to inject effector proteins into eukaryotic cells (Ghosh, 2004). The injected proteins function to disrupt host signaling pathways in a manner that benefits the pathogen. T3SSs, at a minimum, consist of a multicomponent secretion apparatus (or injectisome) that spans the bacterial inner and outer membranes, translocon proteins that assemble a pore-like structure in the eukaryotic membrane, anti-host effector proteins, type III secretion (T3S) chaperones, and a variety of regulatory proteins that control the assembly of the secretion apparatus and the selection of T3S substrates (Hueck, 1998).

All three human pathogenic yersiniae (*Yersinia pestis*, *Y. pseudotuberculosis*, and *Y. enterocolitica*) carry a ca. 70 kb plasmid encoding a T3SS that is required for virulence (Ben-Gurion and Shafferman, 1981; Ferber and Brubaker, 1981; Cornelis et al., 1998). These T3SSs function to inject at least five effector proteins, termed *Yersinia* outer proteins (Yops) into targeted eukaryotic cells (Trosky et al., 2008). The injected effector proteins act to block bacterial phagocytosis or suppress the production of pro-inflammatory cytokines. These activities allow the yersiniae to survive and multiply in the extracellular milieu of their hosts.

Assembly of the T3S apparatus requires the participation of at least 21 *Yersinia* secretion (Ysc) proteins [reviewed in Cornelis and Van Gijsegem (2000)]. The assembled T3S apparatus initially secretes YscI and YscF that assemble an internal rod-like structure and an extracellular needle-like structure, respectively (Edqvist et al., 2003; Wood et al., 2008). The secreted YscP protein serves as a molecular ruler that directs a YscU-dependent substrate specificity switch from needle-type substrates (YscF, YscI, and YscP) to translocator- and/or effector-type substrates when the needle reaches the proper length (Journet et al., 2003; Agrain et al., 2005; Sorg et al., 2007; Wood et al., 2008). At this point, a complex composed of the YopN, SycN, YscB, and TyeA proteins is targeted to the injectisome and functions to prevent Yop secretion until a secretion triggering signal is encountered (Day and Plano, 1998; Ferracci et al., 2005). Importantly, the YopN/SycN/YscB/TyeA-dependent block in Yop secretion can only be established in environments that contain millimolar levels of extracellular calcium (≥1 mM) for instance the blood or extracellular milieu of a mammalian host. *In vitro*, Yop secretion is triggered in the absence of calcium, whereas *in vivo*, Yop secretion is initiated upon contact between a bacterium and a eukaryotic cell (Rosqvist et al., 1994). Importantly, proteins homologous to YopN and TyeA (YopN/MxiC/InvE family proteins) are present in essentially all T3SSs and function to prevent effector protein secretion prior to receiving a secretion activation signal (Pallen et al., 2005). Contact with a host cell membrane serves as an activation signal for many T3SSs; however, other T3SS-specific activation signals have been identified including the dye Congo Red (Bahrani et al., 1997) and alterations in pH (Yu et al., 2010).

YopN is a 293 residue secreted and translocated protein that interacts with the heterodimeric SycN/YscB chaperone via an N-terminal chaperone-binding domain (CBD; residues 32–76) and with TyeA via a C-terminal TyeA-binding domain (TBD) (Schubot et al., 2005). The SycN/YscB chaperone and TyeA are both required for the calcium-dependent regulation of the T3S process; however, while the SycN/YscB chaperone promotes efficient YopN secretion and translocation, TyeA functions to suppress YopN translocation (Cheng et al., 2001; Day et al., 2003). TyeA is believed to mediate a binding event that physically arrests the YopN/TyeA complex at the T3S apparatus in a position that blocks Yop secretion. Activation of the T3S process triggers YopN secretion and removes the block in secretion. Collectively, the YopN/SycN/YscB/TyeA complex is thought to function as a molecular plug that functions to control the access of T3S effectors and translocators to the T3S apparatus.

Proteins homologous to YopN and TyeA are present in essentially all non-flagellar T3SSs; however, in many T3SSs domains homologous to YopN and TyeA are present in a single protein (Pallen et al., 2005; Silva-Herzog et al., 2011). In contrast, proteins homologous to SycN or YscB are present in only a small subset of T3SSs. Interestingly, a large region of the YopN CBD is conserved among all YopN/InvE/MxiC family proteins regardless of the presence or absence of an identified cognate chaperone (Pallen et al., 2005; Younis et al., 2010). This is true even for the *Salmonella enterica* InvE protein, which is not secreted and has no identified cognate chaperone (Kubori and Galan, 2002). The CBDs of numerous effector proteins have been shown to either contain, or overlap with, peptide sequences implicated in diverse functions unrelated to the secretion or translocation of the effector. For example, the CBD of YpkA (YopO), YopE, and YopT each contain a membrane localization domain that is masked within the bacterial cell by the appropriate chaperone (Letzelter et al., 2006). Similarly, the CBD of YopH binds SycH within the bacterial cell and tyrosine-phosphorylated target proteins within the host cell (Montagna et al., 2001). To begin to investigate the role of the YopN CBD in YopN function, we used site-directed mutagenesis to identify residues required for YopN secretion and/or the regulation of Yop secretion.

## Materials and methods

### Bacterial strains and growth conditions

*Y. pestis* and *Escherichia coli* strains used in this study are listed in Table 1. All *Y. pestis* strains used in this study carry a deletion of the *pgm* locus (*pgm*-) and thus, are avirulent via peripheral routes of infection (Une and Brubaker, 1984). *E. coli* and *Y. pestis* strains were routinely grown in heart infusion broth (HIB) or on tryptose blood agar (TBA) plates (Difco Laboratories) at 37°C or 27°C, respectively. For growth and secretion assays *Y. pestis* strains were grown in thoroughly modified Higuchi's (TMH) medium (Goguen et al., 1984) overnight at 27°C, and diluted the next day to an optical density at 620 nm (OD_620_) of 0.20 in 2 ml of fresh media with or without 2.5 mM CaCl_2_ unless otherwise stated. Cultures were grown for 1 h at 27°C then shifted to 37°C for 5 h of growth. One shot® TOP10 chemically competent *E. coli* (Invitrogen Life Technologies) or *E. coli* DH5α were used for routine cloning experiments. Bacteria with resistance markers were cultured in the presence of the appropriate antibiotic(s) at a final concentration of 25 μg/ml (chloramphenicol and kanamycin) or 50 μg/ml (ampicillin and streptomycin).

**Table 1:** **Bacterial strains used in this study**.

**Strain**	**Relevant characteristics^b^**	**Source**
***Y. pestis*^a^**		
KIM8.P39 (parent)	pCD1 (Δ*sycE-yopE*::km), pPCP1^−^, pMT1	Torruellas et al., 2005
KIM8.P62 (Δ*yopE* Δ*yopN*)	pCD1 (Δ*sycE-yopE*::km Δ*yopN*) pPCP1^−^, pMT1	Torruellas et al., 2005
KIM8-3001.P71 (Δ*yopNtyeA*)	pCD1 (Δ*sycE-yopE*::km Δ*yopNtyeA*) pPCP1^−^, pMT1	Ferracci et al., 2004
KIM8-3001.P1 (Δ*yscB*)	pCD1 (Δ*yscB*) pPCP1^−^, pMT1	Jackson et al., 1998
KIM8-3001.PF1 (Δ*sycN* Δ*yscB*)	pCD1 (Δ*sycE-yopE::km sycN yscB*) pPCP1^−^, pMT1	Day and Plano, 1998
KIM8.PS2 (Δ*yopNtyeAsycN* Δ*yscB*)	Sm^r^pCD1 (ΔsycE-*yopE*::dhfr Δ*yopN tyeAsycN* Δ*yscB*) pPCP1^−^, pMT1	This study
***E. coli***		
DH5α	F-Φ80 d *lacZ* ΔM15, Δ(*lacZYA argF*)U189, *endA1 recA1 hsdR17 deoR supE44 thi-1 gyrA96 relA1*	Cambau et al., 1993
Top10	F-mcrA Δ*(mrr-hsdRMS-mcrBC) ϕ80lacZΔM15* Δ*lacX74 nupG recA1 araD139* Δ*(ara-leu)7697 galE15 galK16 rpsL(Str^R^) endA1 λ^−^*	Invitrogen Life Technologies

a *All Y. pestis strains are avirulent due to a deletion of the pgm locus (Une and Brubaker, 1984)*.

b *Plasmids native to Y. pestis include pCD1 (Perry et al., 1998), pPCP1 (Sodeinde et al., 1988) that encodes the outer membrane plasminogen activator protease (Pla) that has been demonstrated to degrade secreted Yops and pMT1 encoding the capsular protein (Protsenko et al., 1983)*.

### Construction of plasmids encoding YopE-YopN hybrid proteins

Plasmids pYopE^1−15^-YopN^16−293^-GSK and pYopE^1−85^-YopN^86−293^-GSK were constructed via the PCR-ligation-PCR technique (Ali and Steinkasserer, 1995). DNA fragments encoding *sycE* and *yopE* amino-acid residues 1–15 as well as *sycE* and *yopE* residues 1–85 were amplified from plasmid pCD1 using upstream primer SycE-F and downstream primers YopE-15R or YopE-85R, respectively (oligonucleotides used in this study are listed in Table 2). The *yopN* sequences encoding amino-acid residues 16-293-GSK and 86-293-GSK were amplified from plasmid pBAD30-YopN-GSK using upstream primers YopN-16F or YopN-86F, respectively and downstream primer YopN-GSK-R. The four resulting PCR fragments were purified, phosphorylated using T4 polynucleotide kinase and ligated to the appropriate DNA fragment with T4 DNA ligase. The ligated products were used as template for a second PCR reaction using the outside primers SycE-F and YopN-GSK-R carrying KpnI and XbaI restriction endonuclease sites, respectively. These fragments were inserted into KpnI- and XbaI-digested pBAD18 (Guzman et al., 1995) generating plasmids pYopE^1−15^-YopN^16−293^-GSK and pYopE^1−85^-YopN^86−293^-GSK. Plasmid pBAD18-YopN-GSK was constructed by excising a YopN-GSK-encoding EcoRI-XbaI fragment from plasmid pBAD30-YopN-GSK (Garcia et al., 2006) and inserting this fragment into EcoRI- and XbaI-digested pBAD18.

**Table 2:** **Oligonucleotides used in this study**.

**Name**	**Sequence^a^**
SycE-F	5′-TTTGGTACCTCAACTAAATGACCGTGGTGGTGA-3′
YopE-15R	5′-TGTCGGCAGGGGCAGTGATGTAGA-3′
YopE-85R	5′-CGGTTTATGGCTCCCTCCGAGAACAT-3′
YopN-16F	5′-AATGAGCGTCCAGAGATTGCCAGTAGT-3′
YopN-86F	5′-CAGGTTAATCAATACCTTAGCAAAGTT-3′
YopN-GSK-R	5′-TTTTCTAGAGAGTCAACTTTCAGCGAAACTAGTAGTGCG-3′
YopN-KpnI-F	5′-TTTGGTACCGAAAAATAGCCAAGC-3′
YopN-NheI	5′-TTTGCTAGCTAAACTGTTCATCTCAGGGAGTAG-3′
TyeA-KpnI	5′-TTTGGTACCGGTTCAATCCAACTCACTCAATTC-3′
YopN-85R	5′-GAGCCTGGCTGTCACTTAATT-3′
YopN-SycN-P1	5′-CCCGCTGCATAATGAGCGTCCAGAGATTGCCAGTAGTCATGTGTAGGCTGGAGCTGCTTC-3′
YopN-SycN-P2	5′-CTGACTTTCCCCTAACCACCCGCTCGCAGCGGTAAAGCCATTGAATATCCTCCTTAGT-3′
YopN-A33-R	5′-GGCCGCCCAGAGTCTGATTTACGATCTG-3′
YopN-A34-F	5′-GCTGCAGGAGAATCTGTGCAGATAGTC-3′
YopN-A37-R	5′-GGCCGCCCGAAATTGACCCAGAGTCTG-3′
YopN-A38-F	5′-GCTGCGCAGATAGTCAGCGGCACTCTG-3′
YopN-A41-R	5′-GGCCGCCACAGATTCTCCCCGAAATTG-3′
YopN-A42-F	5′-GCCGCCGGCACTCTGCAGTCTATAGCT-3′
YopN-A45-R	5′-GGCCGCGCTGACTATCTGCACAGATTC-3′
YopN-A46-F	5′-GCGGCGTCTATAGCTGATATGGCAGAA-3′
YopN-A49-R	5′-GGCCGCCTGCAGAGTGCCGCTGACTAT-3′
YopN-A50-F	5′-GCTGCTATGGCAGAAGAGGTAACATTT-3′
YopN-A53-R	5′-GGCCGCATCAGCTATAGACTGCAGAGT-3′
YopN-A54-F	5′-GCAGCGGTAACATTTGTCTTCTCCGAGA-3′
YopN-A57-R	5′-GGCCGCCTCTTCTGCCATATCAGCTAT-3′
YopN-A58-F	5′-GCTGCCTTCTCCGAGCGTAAGGAGCTC-3′
YopN-A61-R	5′-GGCCGCGACAAATGTTACCTCTTCTGC-3′
YopN-A62-F	5′-GCGGCTAAGGAGCTCTCCCTCGACAAA-3′
YopN-A65-R	5′-GGCCGCACGCTCGGAGAAGACAAATGT-3′
YopN-A66-F	5′-GCCGCCCTCGACAAACGCAAATTAAGT-3′
YopN-A69-R	5′-GGCCGCGGAGAGCTCCTTACGCTCGGA-3′
YopN-A70-F	5′-GCAGCCAAATTAAGTGACAGCCAGGCT-3′
YopN-A73-R	5′-GGCCGCGCGTTTGTCGAGGGAGAGCTC-3′
YopN-A74-F	5′-GCTGCCAGCCAGGCTCGAGTTAGCGAC-3′

a *Restriction enzyme recognition sites are underlined*.

### Generation of plasmids encoding YopN CBD tetra-alanine substitution mutants

Plasmid pBAD30-YopN-GSK encodes a full length fully functional YopN protein with a C-terminal GSK tag (Garcia et al., 2006). Tetra-alanine scanning mutagenesis of *yopN* from pBAD30-YopN-GSK was performed via oligonucleotide-directed mutagenesis in combination with Gateway cloning technology (Invitrogen Life Technologies). Mutation of four consecutive codons encoding YopN CBD residues to codons encoding four alanine residues were generated using the PCR-ligation-PCR technique (Ali and Steinkasserer, 1995) using outside primers YopN-KpnI-F and downstream primer YopN-GSK-R. Inside primers were created to generate four alanine substitutions at the desired positions of the *yopN* sequence. For example to generate YopN(G32-R35A); primers YopN-KpnI-F and YopN-A33R were used to create an initial DNA fragment (fragment A). Primers YopN-A34F and YopN-GSK-R were used to generate a second DNA fragment (fragment B). The resultant DNA fragments (A and B) were purified from agarose gels, phosphorylated using T4 polynucleotide kinase, and ligated using T4 DNA ligase. A final PCR product was generated using the ligation reaction as template with outside primers YopN-KpnI-F and YopN-GSK-R. The final PCR product was inserted into the pCR8/GW/TOPO vector using the pCR8/GW/TOPO TA cloning kit (Invitrogen) and the insert sequence confirmed via DNA sequencing. The correct DNA fragment was inserted into a gateway compatible pBAD30 vector using Gateway LR Clonase II mix (Invitrogen). This procedure was repeated to generate plasmids pBAD30-YopN(G36-V39A)-GSK, pBAD30-YopN(Q40-S43)-GSK, pBAD30-YopN(G44-Q47A)-GSK, pBAD30-YopN(S48-D51A)-GSK, pBAD30-YopN(M52-E55A)-GSK, pBAD30-YopN(V56-V59A)-GSK, pBAD30-YopN(F60-R63A)-GSK, pBAD30-YopN(K64-S67A)-GSK, pBAD30-YopN(L68-R71A)-GSK, and pBAD30-YopN(K72-D75A)-GSK encoding each of the YopN CBD tetra-alanine substitution mutants.

### Generation of pBAD18-YopN/TyeA plasmids

Plasmid pBAD18-YopN/TyeA encodes full length YopN and TyeA under the control of the P_BAD_ promoter of plasmid pBAD18-Cm (Guzman et al., 1995). A DNA fragment encoding YopN and TyeA was amplified from plasmid pCD1 using primers YopN-NheI and downstream primer TyeA-KpnI harboring NheI and KpnI sites, respectively. The DNA fragment was digested with NheI and KpnI and ligated to NheI- and KpnI-digested pBAD18-Cm. The vector carrying the correct DNA insert was confirmed by DNA sequence analysis (Genewiz). Plasmids pBAD18-YopN(S48-D51A)/TyeA, pBAD18-YopN(M52-E55A)/TyeA, pBAD18-YopN(V56-V59A)/TyeA, pBAD18-YopN(F60-R63A)/TyeA, pBAD18-YopN(K64-S67A)/TyeA, pBAD18-YopN(L68-R71A)/TyeA, and pBAD18-YopN(K72-D75A)/TyeA were generated using the PCR-ligation-PCR technique. DNA fragments encoding the respective YopN CBD tetra-alanine substitutions were amplified from plasmids pBAD30-YopN(S48-D51A)-GSK, pBAD30-YopN(M52-E55A)-GSK, pBAD30-YopN(V56-V59A)-GSK, pBAD30-YopN(F60-R63A)-GSK, pBAD30-YopN(K64-S67A)-GSK, pBAD30-YopN(L68-R71A)-GSK, and pBAD30-YopN(K72-D75A)-GSK using primers YopN-NheI and YopN-85R. A second fragment was amplified from plasmid pBAD18-YopN/TyeA using primers YopN-86-F and TyeA-KpnI. The two fragments were agarose gel purified, phosphorylated, and ligated to each other. The resultant DNA fragments were digested with NheI and KpnI restriction endonucleases and ligated to NheI- and KpnI-digested pBAD18-Cm, generating plasmids pBAD18-YopN(S48-D51A)/TyeA, pBAD18-YopN(M52-E55A)/TyeA, pBAD18-YopN(V56-V59A)/TyeA, pBAD18-YopN(F60-R63A)/TyeA, pBAD18-YopN(K64-S67A)/TyeA, pBAD18-YopN(L68-R71A)/TyeA, and pBAD18-YopN(K72-D75A)/TyeA.

### Construction of A *Y. pestis yopNtyeAsycN yscB* deletion strain

Deletion of the *yopN*, *tyeA*, and *sycN* genes from a *Y. pestis yscB* deletion strain (KIM8-3001.PF1) (Jackson et al., 1998) and insertion of a *kan* gene was accomplished using lambda Red-mediated recombination essentially as described by Datsenko and Wanner (2000). The PCR product used to construct the gene replacement was amplified from plasmid pKD4 using primers YopN-SycN-P1 and YopN-SycN-P2. The PCR product was gel purified, ethanol precipitated, and resuspended in 10 μl of dH_2_O. Plasmid pKD46 encoding the Red recombinase was electroporated into the *yscB* deletion strain and grown in HIB for 2 h with 0.2% L-arabinose. Electrocomptent cells were prepared as previously described (Jackson et al., 2004) and electroporated with the purified PCR product. Gene replacements were confirmed by PCR analysis. Plasmid pCP20 (Cherepanov and Wackernagel, 1995) that encodes the FLP recombinase was electroporated into the kanamycin resistant strain to remove the FLP recognition target-flanked *kan* cassette. Plasmid pKD46 and pPCP20 were cured from the *Y. pestis yopNtyeAsycN yscB* deletion mutant (KIM8-3001.PS2) by overnight growth at 39°C.

### Cross-linking and immunoprecipitation of YopN, SycN, YscB, and TyeA

The Δ*yopN* Δ*yopE Y. pestis* strains transformed with pBAD30-YopN-GSK or the pBAD30-YopN-GSK derivatives encoding the YopN CBD tetra-alanine substitution mutants were grown in the absence of calcium at 37°C for 3 h. The immunoprecipitation procedure was adapted from the method of Day and Plano (1998). Bacterial cells from 20 ml cultures were harvested at an OD_620_ = 1.2, resuspended in 3 ml of 20 mM Hepes, 250 mM NaCl, pH 7.4, and lysed by passage through a French pressure cell. Triton X-100 (0.5% final concentration) and SDS (0.1% final concentration) were added to the lysates and the solubilized lysates were centrifuged at 10,000 × g for 10 min at 4°C. Soluble fractions from the strains were cross-linked with the thio-cleavable amine-reactive cross-linker DSP (Thermo Scientific) for 20 min at room temperature at a final concentration of 1 mM. Samples were immunoprecipitated overnight with 20 μl polyclonal antiserum specific for YopN. Antigen-antibody complexes were collected by the addition of 80 μl of 40% (w/v) EZview Red Protein A Affinity Gel (Sigma) that was resuspended in immunoprecipitation buffer (20 mM Hepes, 250 mM NaCl, 0.5% Triton X-100, 0.1% SDS, pH 7.4) and rotated at 4°C for 2 h. Protein-A Sepharose antigen-antibody complexes were collected by centrifugation at 8200 × g for 30 s at room temperature. The samples were washed three times with 1 ml of immunoprecipitation buffer, eluted with 100 μl electrophoresis buffer containing 5% β ME (cleaves the DSP cross-links), boiled for 3 min and analyzed by SDS-PAGE and immunoblotting.

### SDS-PAGE and immunoblotting

Volumes of cellular fractions corresponding to equal numbers of bacteria were mixed 1:1 (v/v) with 2X electrophoresis sample buffer and analyzed by SDS-PAGE and immunoblot analysis as described previously (Ferracci et al., 2005) with rabbit polyclonal antisera specific for YopE, YopM, YopN, SycN, YscB, TyeA, or YscW (Plano and Straley, 1995; Day and Plano, 1998). Proteins were visualized using an alkaline phosphatase-conjugated secondary antibody (Sigma) and 1-Step NBT/BCIP Development Solution (Thermo Scientific) according to the Manufacturer's directions.

## Results

### Role of the YopN CBD in the regulation of Yop secretion

**Figure 1:**
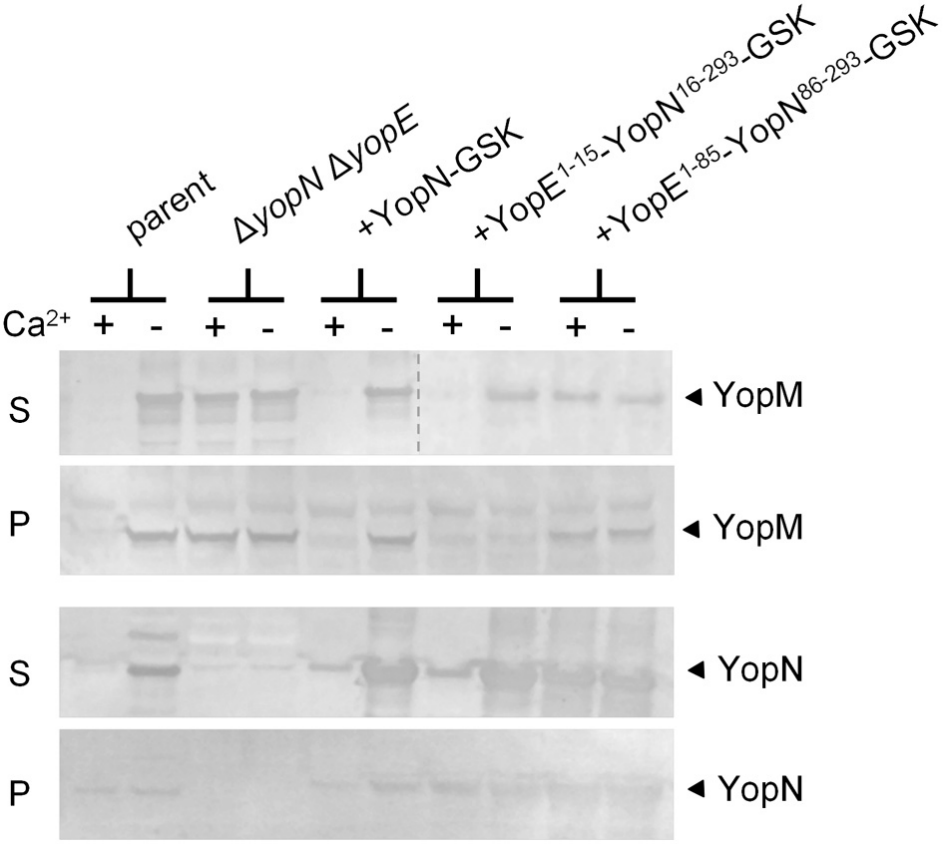
**The YopN CBD but not the N-terminal secretion signal is required to regulate Yop secretion.** Immunoblot analysis of culture supernatant (S) and cell pellet (P) fractions of *Y. pestis* KIM8.P39 (parent), KIM8.P62 (Δ*yopN* Δyop*E*), and KIM8.P62 expressing YopN-GSK, YopE^1−15^-YopN^16−293^-GSK or YopE^1−85^-YopN^86−293^-GSK grown in the presence (+) or absence (−) of 2.5 mM calcium for 5 h at 37°C. Antisera specific for YopN or YopM were used to detect the expression and secretion of the respective proteins. The dashed line indicates the margins of two blots shown in a merged image.

YopN secretion is dependent on an N-terminal secretion signal (SS; residues 2–15), a CBD (residues 32–76), and the SycN/YscB chaperone (Day and Plano, 1998; Goss et al., 2004). To begin to investigate the role of the YopN SS and CBD in the regulation of Yop secretion, we replaced the YopN SS or SS and CBD with the analogous regions of YopE (Woestyn et al., 1996; Lloyd et al., 2001) to determine if the YopN N-terminus or SycN/YscB chaperone has a unique role in the regulation of Yop secretion. YopE is a secreted effector protein with no direct role in the calcium-dependent regulation of the T3S process. Constructs encoding YopN-GSK (pBAD18-YopN-GSK), YopE^1−15^-YopN^16−293^-GSK, and YopE^1−85^-YopN^86−293^-GSK were generated and moved into a Δ*yopE* Δ*yopN Y. pestis* strain. Sequences encoding a C-terminal GSK-epitope tag (Garcia et al., 2006), which does not affect YopN secretion or function, were included in each of the constructs. In addition, sequences encoding the YopE-specific chaperone SycE (Wattiau and Cornelis, 1993) were included in the constructs encoding the two YopE-YopN hybrid proteins. To determine if the hybrid proteins were expressed, secreted, and functional, the strains were grown at 37°C in the presence and absence of 2.5 mM calcium and bacterial pellet and culture supernatant fractions were analyzed by SDS-PAGE and immunoblotting with antibodies specific for YopN and YopM (Figure 1). The parent strain (KIM8.P39) and the Δ*yopE* Δ*yopN* strain complemented with plasmids encoding YopN-GSK or YopE^1−15^-YopN^16−293^-GSK secreted YopM and YopN in the absence but not in the presence of calcium, confirming that the YopN SS has no unique role in the regulation of secretion and can be functionally replaced with the YopE SS. In contrast, the strain expressing YopE^1−85^-YopN^86−293^-GSK secreted YopM and YopN in both the presence and absence of calcium, indicating that the YopN CBD has a role in the regulation of Yop secretion that cannot be complemented by the YopE CBD and SycE chaperone. Importantly, both YopE-YopN hybrid proteins were highly expressed and efficiently secreted in the absence of calcium, suggesting that the defect in the regulation of secretion was not due to a defect in secretion *per se*.

### Site-directed mutagenesis of the YopN CBD

**Figure 2:**
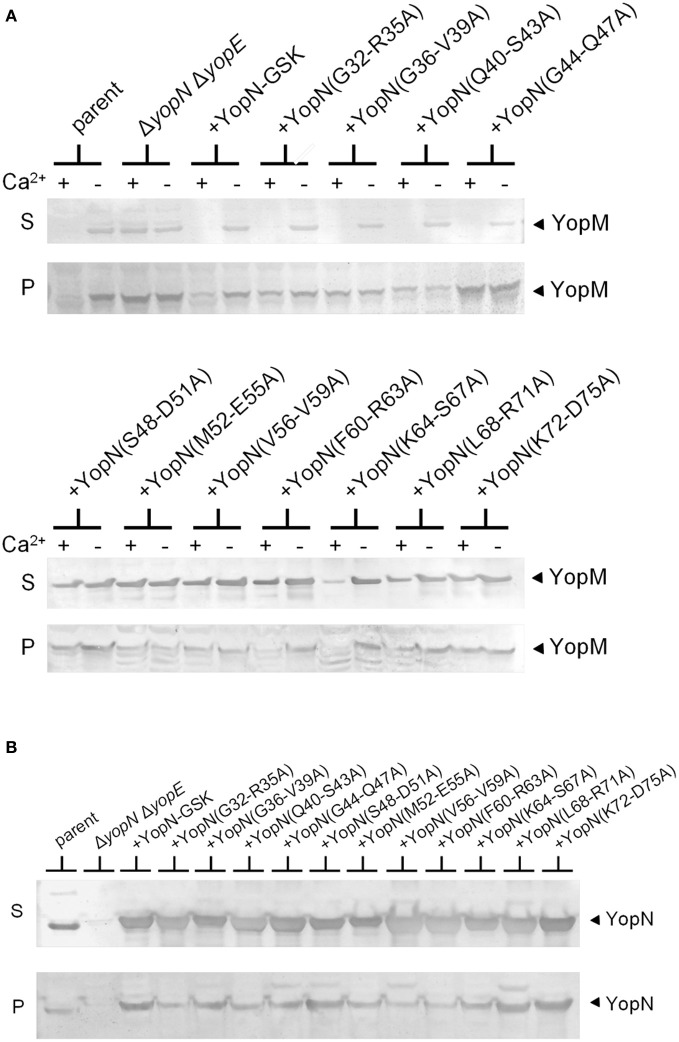
**Residues within the YopN CBD are required to regulate Yop secretion**. A series of tetra-alanine substitution mutants were constructed within YopN CBD residues 32–75. **(A)** Expression and secretion of YopM by *Y. pestis* strains expressing YopN CBD tetra-alanine mutants. KIM8.P39 (Δ*yopE* parent), KIM8.P62 (Δ*yopN* Δ*yopE*), and KIM8.P62 transformed with either pBAD30-YopN-GSK or one of eleven pBAD30-YopN-GSK derivatives encoding YopN CBD tetra-alanine substitution mutants were grown for 5 h at 37°C in the presence (+) and absence (−) of 2.5 mM calcium. Culture supernatant (S) and cell pellet (P) fractions were analyzed by SDS-PAGE and immunoblot analysis with antiserum specific for YopM. **(B)** Expression (P) and secretion (S) of YopN and the YopN tetra-alanine substitution mutants by *Y. pestis* strains grown at 37°C in the absence of calcium. YopN was detected via immunoblot analysis with antiserum specific for YopN.

**Figure 3:**
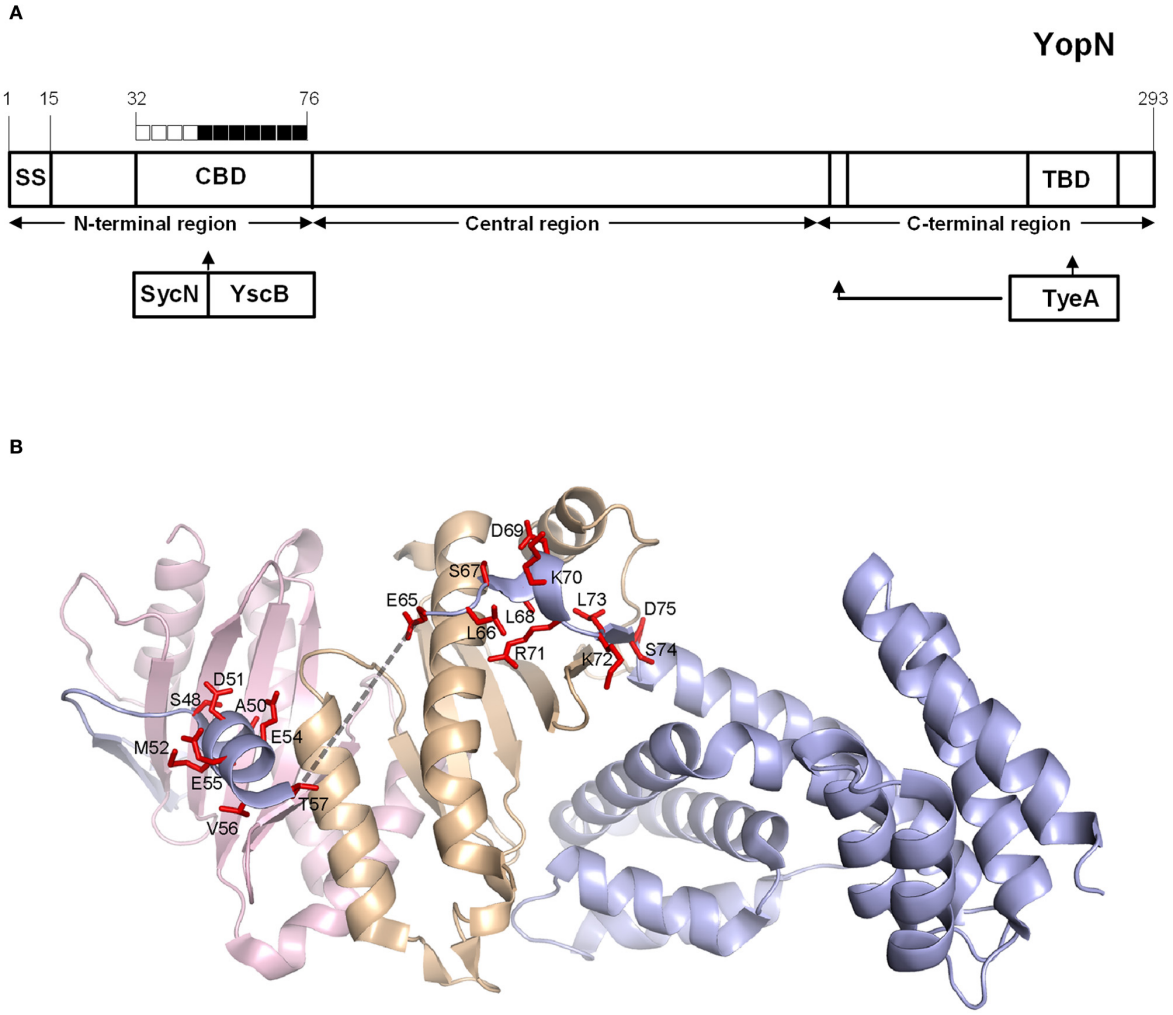
**Mapping of YopN CBD residues involved in the regulation of Yop secretion. (A)** Diagram of YopN showing the location of the secretion signal (SS), chaperone-binding domain (CBD), and TyeA-binding domain (TBD). The extended arrow identifies additional residues contacted by TyeA. Boxes above YopN each represent four YopN residues substituted with four alanines in each YopN tetra-alanine mutant analyzed: open boxes = wild-type calcium-dependent Yop secretion; filled boxes = constitutive Yop secretion. **(B)** Ribbon model of the ternary complex of YopN and the SycN/YscB chaperone. SycN, YscB, and YopN are represented in magenta, brown, and blue, respectively. The side chains of residues of YopN that resulted in constitutive Yop secretion when substituted to alanine are shown in red. The broken line represents the disordered region in the CBD (Schubot et al., 2005). This Figure was generated by PYMOL (http://www.pymol.org).

To further investigate the role of the YopN CBD in the regulation of Yop secretion, a series of tetra-alanine substitution mutants (XXXX → AAAA) were constructed throughout the sequence encoding the CBD (residues 32–76) of plasmid pBAD30-YopN-GSK (Garcia et al., 2006). In total, 11 tetra-alanine substitution mutants were generated, transformed into *Y. pestis* KIM8.P62 (Δ*yopE* Δ*yopN*) and assessed by growth and secretion assays (Figure 2). All of the CBD tetra-alanine substitution mutants were stably expressed and efficiently secreted in the absence of calcium (Figure 2B). YopN CBD mutants G32-R35A, G36-V39A, Q40-S43A, and G44-Q47A showed normal calcium-regulated secretion of YopM (Figure 2A). These mutants specifically altered the CBD region that directly interacts with SycN (Schubot et al., 2005) (Figure 3). In contrast, all of the tetra-alanine substitution mutants, except the YopN(K64-S67A) mutant, that spanned YopN CBD residues 48–75, the CBD region that interacts with YscB (Schubot et al., 2005), secreted YopM in both the presence and absence of calcium, demonstrating that this region of the CBD is required for the regulation of Yop secretion (Figure 2B). The YopN(K64-S67A) mutant exhibited an intermediate phenotype and secreted only low levels of YopM in the presence of calcium. As expected, the YopN CBD mutants that secreted YopM in the presence of calcium also secreted YopN in the presence of calcium (data not shown). Interestingly, the region of the CBD that interacts with SycN is not conserved among YopN/InvE/MxiC family members; in contrast, the CBD region that interacts with YscB and that is essential for the regulation of secretion is well-conserved among YopN/InvE/MxiC family members regardless if they express a YopN-specific chaperone (Pallen et al., 2005) suggesting that this region may have a conserved function in the regulation of the T3S process.

### Interaction of the SycN/YscB chaperone with YopN CBD mutants

**Figure 4:**
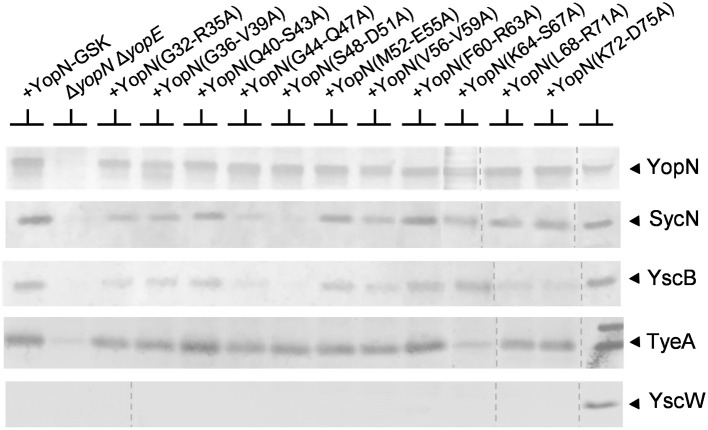
**Interaction of the SycN/YscB chaperone and TyeA with YopN and the YopN tetra-alanine substitution mutants**. The interaction of YopN with the SycN/YscB chaperone was analyzed via immunoprecipitation of YopN and co-immunoprecipitation of SycN and YscB. Soluble lysates of *Y. pestis* KIM8.P39 (parent), KIM8.P62 (Δ*yopN* Δ*yopE*), and KIM8.P62 expressing YopN-GSK or one of the 11 YopN-GSK tetra-alanine substitution mutants grown at 37°C in the absence of calcium were cross-linked with DSP (1 mM), a thio-cleavable amine-specific cross-linking reagent. Antiserum specific for YopN was used to immunoprecipitate YopN, each YopN CBD tetra-alanine mutant and cross-linked SycN/YscB and/or TyeA. Precipitated and co-precipitated proteins were boiled for 5 min in sample buffer containing 5% β-mercaptoethanol to break chemical cross-links and the resulting samples were analyzed by SDS-PAGE and immunoblot analysis with anti-sera specific for YopN, SycN, YscB TyeA, or YscW (control). The dashed lines indicate the margins of individual blots shown in the merged images.

The SycN/YscB chaperone is required for efficient YopN secretion and for the regulation of Yop secretion (Day and Plano, 1998); thus, CBD mutants defective in the regulation of Yop secretion may alter the interaction between YopN and the SycN/YscB chaperone. The interaction of the various YopN CBD tetra-alanine substitution mutants with SycN, YscB, and TyeA was investigated using dithiobis-succinimidyl propionate (DSP) cross-linking and immunoprecipitation (Figure 4). Antisera specific for YopN precipitated YopN from all of the strains expressing either YopN or a YopN CBD mutant. As expected, TyeA, but not the lipoprotein chaperone YscW, co-precipitated with all the YopN CBD mutants; although YopN(K64-S67A) appeared to bind reduced levels of TyeA compared to the other YopN proteins. The SycN/YscB chaperone also efficiently co-precipitated with the YopN CBD mutants with the exception of YopN(G44-Q47A) and YopN(S48-D51A). The SycN/YscB chaperone only binds to YopN as a heterodimer (Day and Plano, 1998); thus, the low amounts of co-precipitating YscB, but normal amounts of SycN, associated with YopN(L68-R71A) and YopN(K72-D75A) are likely due to the loss of critical lysine residues (K70A or K72A) required for DSP-dependent cross-linking of these mutant YopN proteins with YscB. These studies indicate that the YopN CBD mutants defective in the regulation of Yop secretion, with the exception of YopN(G44-Q47A) and YopN(S48-D51A), still bind the SycN/YscB chaperone; however, it is possible that a subset of these mutants exhibit subtle, but possibly significant, alterations in SycN/YscB chaperone binding that cannot be differentiated via DSP cross-linking and immunoprecipitation.

### Role of the SycN/YscB chaperone in the regulation of Yop secretion

**Figure 5:**
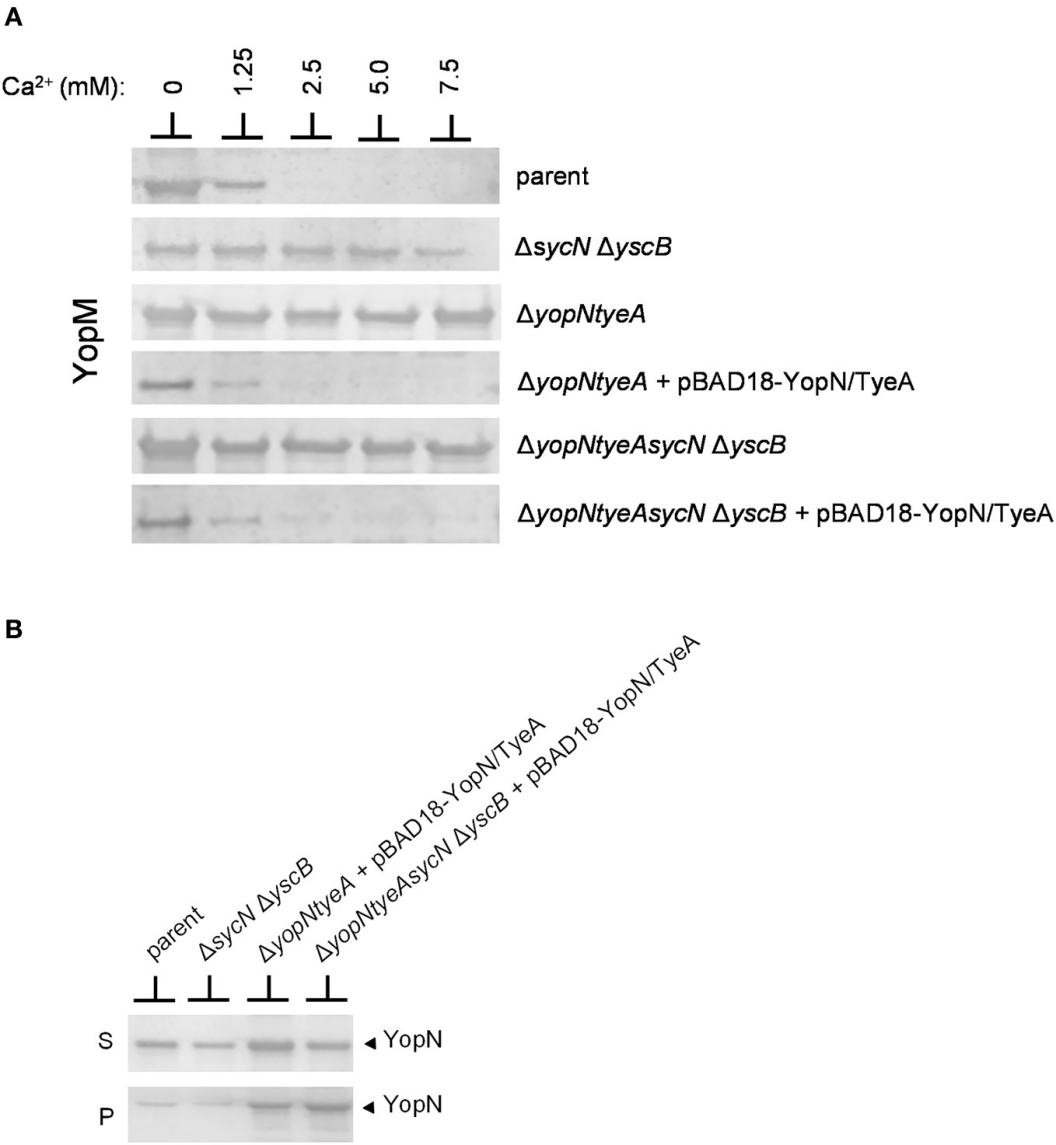
**Overexpression of YopN and TyeA results in increased secretion of YopN and negates the requirement of the SycN/YscB chaperone in the calcium-dependent regulation of Yop secretion. (A)** Secretion of YopM by *Y. pestis* KIM8-3001.P39 (parent), KIM8-3001.PF1 (Δ*sycN* Δ*yscB*), KIM8-3001.P71 (Δ*yopN* Δ*tyeA*), or KIM8.PS2 (Δ*yopN* Δ*sycN* Δ*yscB* Δ*tyeA*) with or without plasmid pBAD18-YopN/TyeA. *Y. pestis* strains were grown for 5 h in the presence of 0 mM, 1.25 mM, 2.5 mM, 5 mM, or 7.5 mM calcium. Expression of YopN and TyeA from plasmid pBAD18-YopN/TyeA was induced with 0.2% L-arabinose. Secreted YopM was detected by immunoblot analysis with antiserum specific for YopM. **(B)** Expression and secretion of endogenous YopN by *Y. pestis* KIM8-3001.P39 (parent) and KIM8-3001.PF1 (Δ*sycN* Δ*yscB*) or plasmid-expressed YopN by *Y. pestis* KIM8-3001.P71 (Δ*yopN* Δ*tyeA*) or KIM8-3001.PS2 (Δ*yopN* Δ*sycN* Δ*yscB* Δ*tyeA*) carrying plasmid pBAD18-YopN/TyeA. Strains with or without plasmid pBAD18-YopN/TyeA were induced with 0.2% L-arabinose and grown for 5 h in the absence of calcium. Bacterial pellet (P) and culture supernatant (S) proteins were separated by SDS-PAGE and subjected to immunoblot analysis with antisera specific for YopN.

Previous studies conducted using *sycN* and/or *yscB* deletion strains have demonstrated that the SycN/YscB chaperone is required for efficient YopN secretion, YopN translocation, and for the regulation of Yop secretion (Day and Plano, 1998; Cheng et al., 2001; Day et al., 2003). The SycN/YscB chaperone is unique among class I T3S chaperones in that it functions as a heterodimer, and thus, it is not unreasonable that it could have a unique and direct role in the regulation of Yop secretion. Conversely, SycN/YscB may simply function to promote Yop secretion, a process that may need to be efficient to regulate Yop secretion. To further investigate the role of the SycN/YscB chaperone in the regulation of Yop secretion, we examined the role of SycN/YscB in the presence of increasing amounts of extracellular calcium and upon overexpression of YopN and TyeA (Figure 5). *Y. pestis* strains lacking *sycN* and *yscB*, *yopN*, and *tyeA* or *yopN*, *tyeA*, *sycN*, and *yscB* secreted YopM in the presence and absence of calcium (Figure 5A). Furthermore, increasing the amount of extracellular calcium did not block or significantly reduce the amount of YopM secreted by these strains. Providing plasmid pBAD18-YopN/TyeA restored normal calcium-dependent regulation of YopM secretion to the *yopN tyeA* deletion strain and, unexpectedly, also to the *yopNtyeAsycN yscB* deletion strain, demonstrating for the first time that calcium-dependent regulation of Yop secretion can be achieved in the absence of the SycN/YscB chaperone. Importantly, the increased expression of YopN from plasmid pBAD18-YopN/TyeA resulted in levels of YopN secretion that exceeded levels secreted by the wild-type parent strain (Figure 5B) even in the absence of the SycN/YscB chaperone. Overall, these results indicate that the defect in the regulation of secretion associated with deletion of *sycN* or *yscB* is due to the inefficient secretion of YopN in the absence of its chaperone, a defect that can be overcome by overexpression of YopN and TyeA.

**Figure 6:**
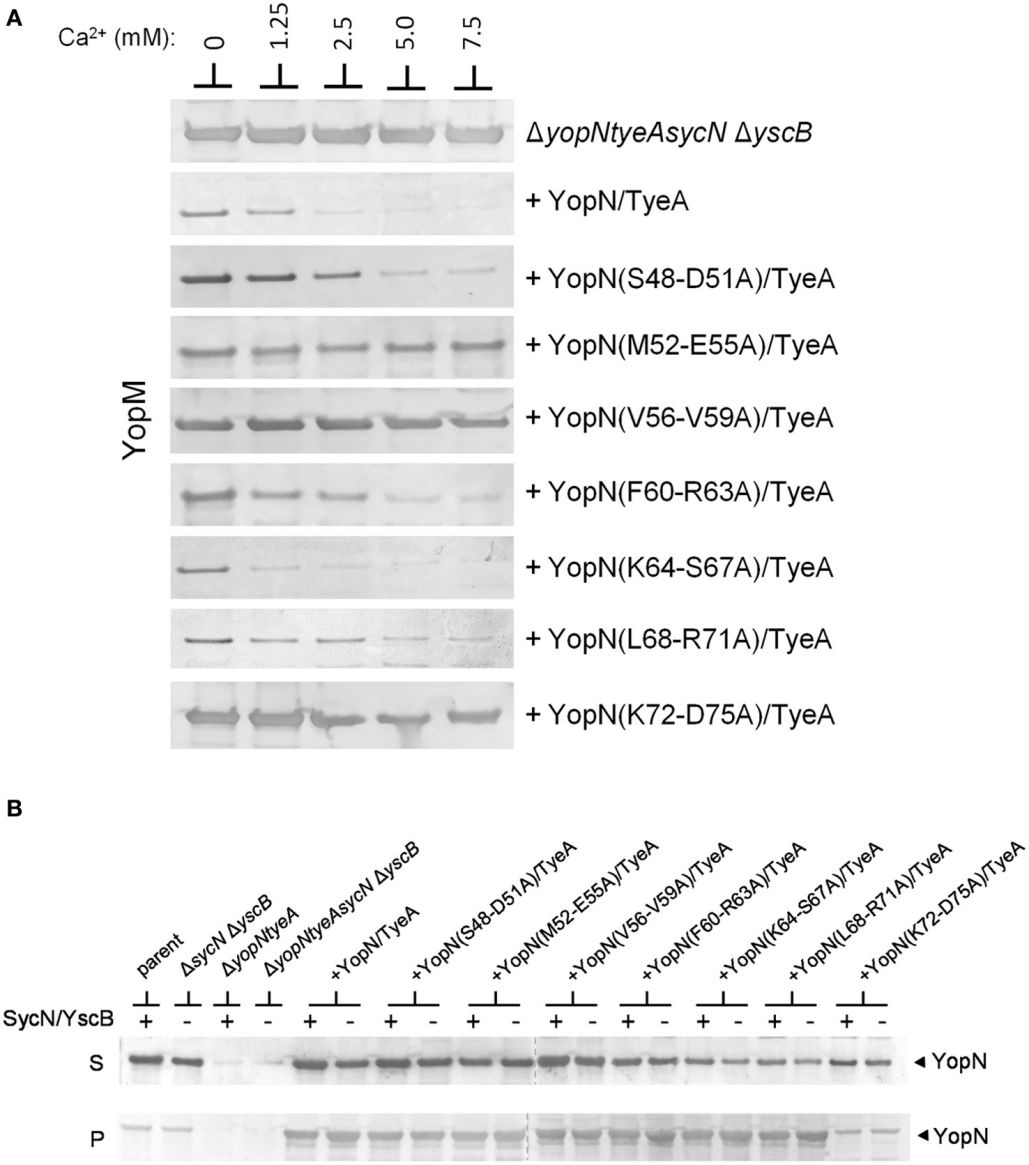
**Secretion of YopM by the YopN CBD tetra-alanine mutants under SycN/YscB chaperone-independent conditions. (A)** *Y. pestis* KIM8-3001.PS2 (Δ*yopN* Δ*sycN* Δ*yscB* Δ*tyeA*) carrying pBAD18-YopN/TyeA or pBAD18-YopN/TyeA derivatives encoding one of the YopN CBD tetra-alanine mutants that previously exhibited a defect in the regulation of Yop secretion under normal SycN/YscB chaperone-dependent conditions were grown for 5 h in the presence of 0 mM, 1.25 mM, 2.5 mM, 5 mM, or 7.5 mM calcium. Expression of YopN, the YopN CBD tetra-alanine substitution mutants and TyeA from plasmid pBAD18-YopN/TyeA or derivatives of this plasmid was induced with 0.2% L-arabinose. **(A)** Secreted YopM was detected by immunoblot analysis with antiserum specific for YopM. **(B)** Expression and secretion of YopN and YopN CBD tetra-alanine substitution mutants by *Y. pestis* KIM8-3001.P71 (Δ*yopN* Δ*tyeA*) (+SycN/YscB) and KIM8.PS2 (Δ*yopN* Δ*sycN* Δ*yscB* Δ*tyeA*) (−SycN/YscB) strains carrying pBAD18-YopN/TyeA or one of the pBAD18-YopN/TyeA derivative encoding the YopN CBD tetra-alanine mutants. Control strains included *Y. pestis* KIM8.P39 (parent) and KIM8-3001.PF1 (Δ*sycN* Δ*yscB*). YopN present in the bacterial pellet (P) and culture supernatant (S) fractions was detected by immunoblot analysis with antiserum specific for YopN. The dashed lines indicate the margins of individual blots shown in the merged images.

The establishment of conditions (overexpression of YopN and TyeA) that enable SycN/YscB-independent regulation of Yop secretion provides an additional means to differentiate whether the constitutive secreting YopN CBD mutants are defective in the regulation of secretion due a chaperone-dependent defect in YopN secretion or to a defect in a novel chaperone-independent function of this region of YopN. Each of the CBD mutants that displayed a defect or partial defect in the regulation of Yop secretion were moved into plasmid pBAD18-YopN/TyeA and transformed into the *yopNtyeAsycN yscB* deletion mutant. The YopN(M52-E55A), YopN(V56-V59A), and YopN(K72-D75) tetra-alanine substitution mutants clearly maintained their constitutive secretion phenotype under conditions where the SycN/YscB chaperone is not required for YopN secretion or the regulation of Yop secretion (Figure 6A). Importantly, each of the YopN mutants was stably expressed and efficiently secreted both in the presence and absence of the SycN/YscB chaperone (Figure 6B). Levels of the YopN(K72-D75) mutant in the cell pellet were lower than the other mutants, suggesting that inefficient expression of this particular mutant could have contributed to the constitutive secretion of YopM in Figure 6A. Overall, these results indicate that the constitutive secretion phenotype associated with the YopN(M52-E55A), YopN(V56-V59A), and possibly the YopN(K72-D75) CBD mutants was not due to a defect in the chaperone-dependent targeting of YopN for secretion, but to a defect in a chaperone-independent function of the CBD region of YopN in the calcium-dependent regulation of Yop secretion.

## Discussion

Previous studies have established that the YopN SS, CBD, and SycN/YscB chaperone are required for efficient YopN secretion and translocation (Day and Plano, 1998; Goss et al., 2004; Garcia et al., 2006). In this study, we examined the role of the YopN SS, CBD, and SycN/YscB chaperone in the calcium-dependent regulation of Yop secretion. The YopN SS could be functionally replaced by the analogous region of YopE, indicating that the YopN SS has no unique role in the regulation of Yop secretion. In contrast, the function of the YopN CBD and SycN/YscB chaperone in the regulation of Yop secretion could not be restored by the YopE CBD and SycE chaperone, suggesting that the YopN CBD or the SycN/YscB chaperone has a direct role in the regulation of Yop secretion. Importantly, the hybrid YopE/YopN proteins were efficiently secreted, indicating that the YopN CBD or SycN/YscB chaperone have a role in the regulation of Yop secretion that is separate from their role in targeting YopN for secretion.

The role of T3S chaperones in the secretion process is not fully understood and can vary depending upon the chaperone/substrate pair (Feldman and Cornelis, 2003). The chaperones for YopN, and closely related homologs that interact with other YopN/InvE/MxiC family proteins, are considered unique as they function as heterodimers (Day and Plano, 1998), suggesting that these chaperone complexes may have a distinctive role in the regulation of Yop secretion. Indeed, *Y. pestis* mutants lacking either SycN or YscB secrete Yops constitutively, confirming a role for these chaperones in the regulation of Yop secretion (Day and Plano, 1998; Jackson et al., 1998). The SycN/YscB chaperone could simply be required to efficiently target YopN to the T3S apparatus; alternatively, SycN, YscB, or both proteins may have a unique and direct role in the regulation of secretion, apart from their role in YopN secretion. Interestingly, the *yscB* gene and genes encoding *yscB* homologs in other T3SSs are located within a multicistronic operon encoding components of the T3S apparatus (*yscBCDEFGHIJKL* operon in the yersiniae) located ca. 9-kb from the *yopN* locus (Perry et al., 1998). Conversely, the gene for *sycN* is encoded directly adjacent to the genes encoding YopN and TyeA (as are the genes encoding *sycN* homologs in other T3SSs), leading to speculation that YscB may mediate a regulation-specific interaction with the T3S apparatus. Here we present evidence that the SycN/YscB chaperone functions, like most other class I T3S chaperones, to efficiently target the YopN/TyeA complex to the T3S apparatus. Overexpression of YopN and TyeA in a strain lacking both the SycN and YscB chaperones restored normal calcium-dependent regulation of Yop secretion, indicating that the SycN/YscB chaperone has no unique and/or indispensable role in the calcium-dependent regulation of Yop secretion. Importantly, other independent lines of evidence also support this contention. First, analysis of the YopN^32−277^/SycN/YscB structure revealed that the SycN/YscB chaperone, although unique, shows a high degree of structural similarity to the homodimeric T3S chaperones, such as SycE (Schubot et al., 2005). Furthermore, YopN/TyeA homologs in other T3SSs, such as MxiC (Botteaux et al., 2009) and InvE (Kubori and Galan, 2002) function in the absence of identified SycN or YscB homologs, indicating again, that the proper regulation of effector protein secretion can occur in the absence of SycN or YscB-type chaperones.

To further analyze the role of the YopN CBD in the regulation of Yop secretion, a series of tetra-alanine substitution mutants within the CBD were constructed. YopN CBD mutants covering residues 32–47, which mediate direct interactions with SycN, were not required for the regulation of Yop secretion (Figure 3); in contrast, tetra-alanine substitutions within residues 48–75, the region that mediates interaction with YscB (Schubot et al., 2005), were required for the regulation of Yop secretion. The majority of these CBD tetra-alanine mutants maintained their ability to bind the SycN/YscB chaperone as demonstrated via immunoprecipitation assays. This suggests that the constitutive secretion phenotype associated with these mutants could be due to the disruption of CBD residues that have a direct function in the regulation of Yop secretion, not to their interaction with the SycN/YscB chaperone. This appears to be particularly true for YopN residues V56–V59, which are part of an unstructured region within the CBD that plays no role in the interaction of YopN with the SycN/YscB chaperone, but does carry amino-acid sequence-specific information required to regulate Yop secretion (Schubot et al., 2005).

To directly determine if the defect in the regulation of secretion associated with specific YopN CBD tetra-alanine mutants was due to a defect in SycN/YscB-dependent secretion or due to a unique chaperone-independent function of the CBD, we evaluated the ability of the seven YopN CBD mutants that secreted Yops constitutively under normal (SycN/YscB chaperone-dependent) conditions to regulate Yop secretion under the established SycN/YscB-independent conditions (overexpression of YopN and TyeA). Two of the mutants [YopN(S48-D51A) and YopN(F60-R63A)] regained some ability to regulate Yop secretion under the SycN/YscB-independent conditions, indicating that the earlier defect in regulation was chaperone-dependent and likely the result of an improper interaction between the mutant YopN proteins and the SycN/YscB chaperone. Indeed, both SycN and YscB failed to immunoprecipitate with the YopN(S48-D51A) mutant. In contrast, both SycN and YscB immunoprecipitated with YopN(F60-R63A), suggesting that the chaperone-dependent defect in YopN function in this mutant was more complex and not due to a simple loss of chaperone binding. Importantly, these types of mutations are expected and support the previously identified role of the SycN/YscB chaperone in efficiently targeting YopN for secretion. In contrast, to chaperone-dependent mutants discussed above, two of the YopN CBD mutants [YopN(M52-E55A) and YopN(V56-V59A)] secreted Yops constitutively under chaperone-independent conditions, indicating that these mutants were likely defective in regulation due to a unique (SycN/YscB-independent) role of the YopN CBD in the regulation of Yop secretion.

What could be the direct role of the YopN CBD in the regulation of Yop secretion? Our current hypothesis proposes that the N-terminal region of YopN (SS and CBD with the associated SycN/YscB chaperone) is required for the efficient targeting of the wild-type YopN/TyeA complex to the T3S apparatus. At the T3S apparatus, the SycN/YscB chaperone may be removed, revealing the CBD. Alternatively, it is possible that the SycN/YscB chaperone remains bound until the secretion of YopN is triggered. We hypothesize that an early interaction mediated via the CBD region is required for the C-terminal region of YopN complexed with TyeA to establish its unique interaction with the T3S apparatus that blocks Yop secretion. This CBD-dependent interaction with the secretion apparatus may be required to induce a conformational change in the YopN/TyeA complex that is required for it to establish its block in Yop secretion. This mechanism would ensure that the YopN/SycN/YscB/TyeA complex would only block the T3S process at the proper time, when the needle assembly is complete and translocator- and effector-type substrates are targeted to the T3S apparatus. We have previously isolated mutations in YopN that allow the YopN/TyeA complex to constitutively block secretion regardless of the level of extracellular calcium (Ferracci et al., 2005). Surprisingly, these mutants no longer required the YopN SS, CBD, or SycN/YscB chaperone to block Yop secretion, confirming that the C-terminal region of YopN complexed with TyeA mediates a unique interaction with the T3S apparatus that blocks Yop secretion. We believe these mutations result in a YopN/TyeA complex that is locked in a blocking conformation and thus, no longer requires interactions mediated via the YopN SS, CBD, and SycN/YscB chaperone to target the YopN/TyeA complex to the T3S apparatus and induce the conformational change normally required to mediate a block in Yop secretion. Confirmation of this model will require identification of the T3SS components that YopN directly interacts with to initiate the block in Yop secretion. A previous study found that the *Shigella flexneri* MxiC protein, a homolog of YopN and TyeA, interacts with Spa47, the (T3S) ATPase (YscN in the *Yersinia* T3SS) (Botteaux et al., 2009). Similarly, the YscN ATPase likely functions in the recognition of the YopN/SycN/YscB/TyeA complex and ultimately to remove the SycN/YscB chaperone (Akeda and Galan, 2005); thus, interactions with the T3SS ATPase could simply represent a conserved step in the T3S secretion process and may or may not play a unique role in the regulation of T3S secretion process. Alternatively, structural analysis of the constitutive blocking YopN mutants and comparisons with the structure of wild-type YopN may provide insight into conformational changes required for YopN to block secretion.

### Conflict of interest statement

The authors declare that the research was conducted in the absence of any commercial or financial relationships that could be construed as a potential conflict of interest.
